# Deep learning reconstruction of zero-echo time sequences to improve visualization of osseous structures and associated pathologies in MRI of cervical spine

**DOI:** 10.1186/s13244-025-01902-0

**Published:** 2025-01-29

**Authors:** Malwina Kaniewska, Fabio Zecca, Carina Obermüller, Falko Ensle, Eva Deininger-Czermak, Maelene Lohezic, Roman Guggenberger

**Affiliations:** 1 Institute of Diagnostic and Interventional Radiology, University Hospital Zurich (USZ), Zurich, Switzerland; 2 University of Zurich (UZH), Zurich, Switzerland; 3 Department of Radiology, University Hospital of Cagliari, Monserrato, Italy; 4 Department of Nuclear Medicine, University Hospital Zurich, Zurich, Switzerland; 5 Department of Forensic Medicine and Imaging, Institute of Forensic Medicine, University of Zurich, Zurich, Switzerland; 6 GE HealthCare, Zurich, Switzerland

**Keywords:** Magnetic resonance imaging, Zero-echo time, Deep learning reconstruction, CT-like MRI, Cervical spine

## Abstract

**Objectives:**

To determine whether deep learning-based reconstructions of zero-echo-time (ZTE-DL) sequences enhance image quality and bone visualization in cervical spine MRI compared to traditional zero-echo-time (ZTE) techniques, and to assess the added value of ZTE-DL sequences alongside standard cervical spine MRI for comprehensive pathology evaluation.

**Methods:**

In this retrospective study, 52 patients underwent cervical spine MRI using ZTE, ZTE-DL, and T2-weighted 3D sequences on a 1.5-Tesla scanner. ZTE-DL sequences were reconstructed from raw data using the AirReconDL algorithm. Three blinded readers independently evaluated image quality, artifacts, and bone delineation on a 5-point Likert scale. Cervical structures and pathologies, including soft tissue and bone components in spinal canal and neural foraminal stenosis, were analyzed. Image quality was quantitatively assessed by signal-to-noise ratio (SNR) and contrast-to-noise ratio (CNR).

**Results:**

Mean image quality scores were 2.0 ± 0.7 for ZTE and 3.2 ± 0.6 for ZTE-DL, with ZTE-DL exhibiting fewer artifacts and superior bone delineation. Significant differences were observed between T2-weighted and ZTE-DL sequences for evaluating intervertebral space, anterior osteophytes, spinal canal, and neural foraminal stenosis (*p* < 0.05), with ZTE-DL providing more accurate assessments. ZTE-DL also showed improved evaluation of the osseous components of neural foraminal stenosis compared to ZTE (*p* < 0.05).

**Conclusions:**

ZTE-DL sequences offer superior image quality and bone visualization compared to ZTE sequences and enhance standard cervical spine MRI in assessing bone involvement in spinal canal and neural foraminal stenosis.

**Critical relevance statement:**

Deep learning-based reconstructions improve zero-echo-time sequences in cervical spine MRI by enhancing image quality and bone visualization. This advancement offers additional insights for assessing bone involvement in spinal canal and neural foraminal stenosis, advancing clinical radiology practice.

**Key Points:**

- Conventional MRI encounters challenges with osseous structures due to low signal-to-noise ratio.
- Zero-echo-time (ZET) sequences offer CT-like images of the C-spine but with lower quality.
- Deep learning reconstructions improve image quality of zero-echo-time sequences.
- ZTE sequences with deep learning reconstructions refine cervical spine osseous pathology assessment.
- These sequences aid assessment of bone involvement in spinal and foraminal stenosis.

**Graphical Abstract:**

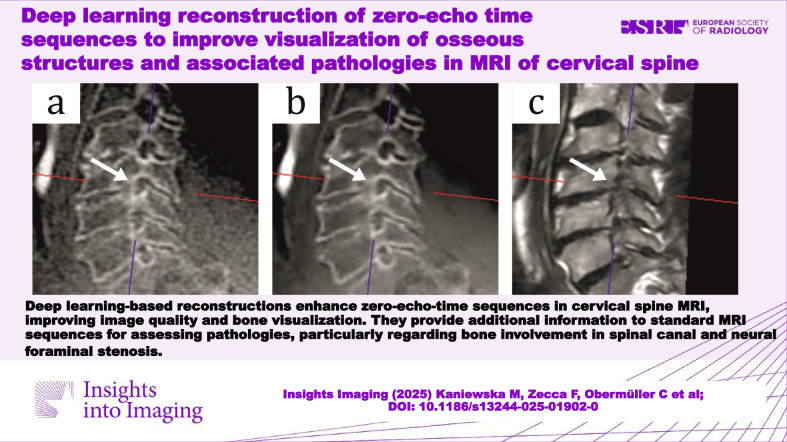

## Introduction

Neck pain is a common problem with over 30% of the general population affected annually [1, 2]. It is the fourth most prevalent contributor to disability among adults, resulting in a substantial amount of various medical examinations performed to define its origin [3, 4].

According to the appropriateness criteria of the American College of Radiology, radiographs are included in the initial evaluation of cervical pain [5]. CT is considered a gold standard for the assessment of osseous structures by providing precise visualization of cortical bone and in evaluating conditions such as osteoarthritis, osteophyte formation, or subchondral sclerosis [6, 7]. MRI is performed in patients with cervical radiculopathy because it provides a detailed evaluation of soft tissues such as nerve roots or intervertebral discs [5]. As cervical pain may be caused by osseous and soft tissue pathologies, it would be desirable to assess both tissue types with a single imaging modality.

However, due to the dense and intricately organized composition of osseous structures with scarcity of mobile protons, conventional MRI sequences provide limited value in their assessment [8]. To account for that, imaging sequences with extremely short repetition times like zero-echo time (ZTE) imaging have been developed and implemented for “CT-like” imaging of spine, shoulder and hip [9–11].

Bone delineation using CT remains superior to the CT-like images from ZTE sequences due to better spatial resolution, signal-to-noise ratio (SNR), and shorter scan times [12, 13]. Recent research has aimed at enhancing SNR efficiency within acceptable scan times by developing methods to retrieve central k-space data missed during the dead-time gaps caused by radiofrequency excitation and switching in ZTE imaging [14, 15]. As the fundamental limitations of conventional image reconstruction remain, different approaches have been introduced, including deep learning-based reconstructions, which provide excellent image quality with substantially reduced scan times [16].

Integrating ZTE sequences with deep learning image reconstruction (ZTE-DL) has the potential to minimize image noise in MRI of the cervical spine, leading to better image quality with improved delineation and evaluation of osseous structures while retaining adequate soft tissue contrast.

Therefore, the aim of this study is to assess whether ZTE-DL sequences can enhance MR imaging of the cervical spine in terms of image quality and bone evaluation, relative to conventional ZTE reconstruction technique and to evaluate the complementary value of DL sequences in conjunction with standard cervical spine MRI for comprehensive pathology assessment.

## Materials and methods

The institutional review board approved this study. Written informed consent was obtained from all participants before imaging.

### Patients

Patients referred by various clinical physicians for MRI of the cervical spine between October 2022 and March 2024 were consecutively included in the study. Patients were excluded if retrospective reconstruction of ZTE-DL sequences was not possible due to incomplete storage of raw data at the time of acquisition, or if they were under 18 years of age.

### MRI protocol and MR acquisition

All MRI examinations were performed using a 1.5-T MRI scanner (SIGNA Artist, GE HealthCare, Waukesha, WI, USA). Patients were positioned supine and scanned with a dedicated 19-channel head and neck coil. Standard MRI of the cervical spine included 3D T2-weighted sequences which were used for assessment of cervical structures. Additionally, 3D isotropic radial ZTE pulse sequences were acquired as part of the study. Detailed scan parameters of the ZTE and T2-weighted sequences are shown in Table 1.

**Table 1:** Scan parameters of ZTE and T2 sequence

Sequence type	ZTE	T2w
MRI acquisition type	3D	3D
Echo time (ms)	0.0	98
Repetition time (ms)	596.7	1402
Slice thickness (mm) (gap, mm)	2.0 (1 mm)	1.4 (0.7 mm)
Acquisition matrix	256 × 256	280 × 280
Flip angle (°)	2	90
Pixel bandwidth (kHz)	390	244
Number of averages	4	2
Field of view (mm)	100	70

ZTE-zero-time echo MRI sequences ZTE sequences were acquired in the sagittal plane and then transferred to a multiplanar viewer. T2-weighted sequences were acquired in the axial plane

### Deep learning reconstruction of ZTE-DL sequences

Initially, the raw ZTE sequences were acquired in the sagittal plane and then transferred to a special post-processing software provided by the vendor for deep learning-based reconstruction known as AIR™ Recon DL (GE HealthCare, Waukesha, WI). These sequences were finally labeled as ZTE-DL sequences.

The AIR™ Recon DL algorithm utilizes a deep convolutional neural network (CNN) that processes raw, complex-valued imaging data to generate a refined output image. The CNN is engineered to allow user-adjustable noise reduction while minimizing truncation artifacts and enhancing edge sharpness. Integration into the scanner’s native, inline reconstruction pipeline is crucial, as it enables access to raw, full bit-depth data. The CNN comprises 4.4 million trainable parameters within approximately 10,000 kernels, making it a convolutional network suitable for all MR-relevant image sizes. The CNN was trained using a supervised learning approach, utilizing pairs of images that represented near-perfect and conventional MRI images. The near-perfect training data consisted of high-resolution images with very low noise levels and hardly any artifacts. Conventional training data were synthesized from these near-perfect images using established methods to create lower-resolution versions with more truncation artifacts and higher noise levels [17].

The dedicated software for image post-processing (Orchestra SDK, GE HealthCare, Waukesha, WI) employs the AIR™ Recon DL algorithm to eliminate noise and Gibbs ringing artifacts from the raw input data before computing the final image. Moreover, the software offers a user-adjustable noise reduction factor ranging from 0 to 100% or categorized as low, medium, or high. Denoising is managed independently of the ringing reduction and does not impact edge sharpness, thereby preserving image features [17].

Prior to the study, a sample set of exams not included in the analysis was reconstructed at different denoising levels and reviewed by the authors. To explore the maximum difference between the reconstruction methods and to identify any potential blurring or loss of image details, the maximal denoising level of 100% was chosen for the ZTE-DL sequences in this study. All image data sets were ultimately transferred to the PACS (DeepUnity Diagn, Dedalus, Bonn, Germany) of our department for further analysis.

### Image analysis

During the initial training phase, a set of ten MRI examinations of the cervical spine, not included in the study sample, were evaluated using ZTE and ZTE-DL sequences in a multiplanar reconstruction mode. Any discrepancies were thoroughly discussed until consensus was reached. Subsequently, ZTE and ZTE-DL images were independently assessed by three readers: a board-certified radiologist with 9 years of experience, and two radiology residents with 4 and 3 years of experience, respectively. All readers were blinded to any clinical information. Additionally, all image sets were anonymized to remove sequence identifiers (ZTE versus ZTE-DL sequences) and then randomized. The reviewers assessed all images in a random order using a multiplanar reconstruction mode in PACS. To ensure a CT-like image impression of ZTE and ZTE-DL sequences, the images were gray-scale inverted for the readout process. Following the assessments, the sequence-type information was disclosed for statistical analysis purposes.

### Qualitative assessment of image quality and osseous structures

The overall image quality of ZTE and ZTE-DL sequences was evaluated using a 5-point Likert scale (0—poor, 1—mild, 2—moderate, 3—good, and 4—perfect). Assessment of osseous structures was assessed separately for cortical bone at the level of the atlantoaxial joint and C5 vertebra and for trabecular bone at the level of C2 and C5 vertebra. The scoring systems for the assessment of image quality and osseous structures are described in Table 2.

**Table 2:** Scoring systems for assessment of image quality and osseous structures

Score	Image quality	Osseous structures
0 (poor)	Very bad image quality	Delineation impossible
1 (mild)	Structures poorly visible, lesion detection hardly possible	Delineation heavily distorted and hardly possible
2 (moderate)	Detectable image noise, preserved delineation	Delineation slightly distorted but possible
3 (good)	Minor image noise, very good depiction of structures	Good delineation with minor distortion
4 (perfect)	Best image quality, perfect depiction	Perfect delineation, no distortion

Artifacts were additionally assessed using a 4-point Likert scale with the following descriptors: 0—mild artifact not obscuring any structures; 1—moderate artifact not obscuring any structures, 2—moderate artifact mildly obscuring, and 3—severe obscuration of structures.

### Quantitative assessment of image quality

To quantitatively evaluate image quality, the signal-to-noise ratio (SNR) and contrast-to-noise ratio (CNR) were measured for ZTE and ZTE-DL sequences. Regions of interest (ROIs) of 5 mm² were placed separately on each image set to determine the signal intensity in bone (at the level of C2 and C7 vertebra) and muscle (trapezius muscle at the level of C6 vertebra).

The mean and standard deviation (SD) values of signal intensity were calculated for each ROI. To estimate noise, the average SD values for both bone and muscle were computed from various anatomical locations as previously described [9].

The mean signal values from both cortical and trabecular bone measurements were used to represent bone signal for the calculation of SNR and CNR, which were determined as follows:

$${SNR}=\frac{{SI}({bone})}{{mean\; SD}({bone\; and\; muscle})}$$

$${CNR}=\frac{{SI}\left({bone}\right)-{SI}({muscle})}{{mean\; SD}({bone\; and\; muscle})}$$

### Cervical structures and associated pathologies

To best represent a routine clinical setting and determine the additional value of ZTE sequences, standard 3D T2-weighted sequences were evaluated alone, and then together with either ZTE or ZTE-DL sequences for the assessment of cervical structures and associated pathologies. Additionally, a subgroup analysis including patients with a previous CT of the cervical spine was performed and compared to the ZTE and ZTE-DL sequences.

Each disc level across the entire cervical spine was evaluated, and the following structures were assessed: intervertebral space (0—normal, 1—mild height loss < 25%, 2—moderate height loss of 25–50%, 3—height loss of 50 to 75% and 4—severe height loss of > 75%), anterior and posterior osteophytes (0—normal, no osteophytes, 1—mild, osteophytes of < 1/8 AP diameter of the corresponding vertebral body, 2—moderate, 1/8–1/4 AP diameter and 3—severe, > 1/4 AP diameter), stenosis of spinal canal (0—normal, 1—mild, < 25% narrowing of the spinal canal, 2—moderate, 25–50% narrowing, and 3—severe, > 50% narrowing) and neural foramen stenosis (0—normal, 1—mild, < 25% narrowing of the neural foramen, 2—moderate, 25–50% narrowing, and 3—severe, > 50% narrowing). Right and left neural foramen were evaluated separately at each analyzed level of the cervical spine.

When spinal canal stenosis was noted, an additional characterization was performed as follows: 0—entirely due to disc, 1—mostly disc, 2—disc = bone, 3—mostly bone and 4—due to bone [18]. Characterization of the neural foraminal stenosis was assessed accordingly (0—entirely due to soft tissue, 1—mostly soft tissue, 2—soft tissue = bone, 3—mostly bone, and 4—due to bone). Each structure was evaluated in a multiplanar tool of the obtained 3D image sets.

### Statistical analysis

All assessments of qualitative and quantitative image quality were consolidated and compared between sequences using a Wilcoxon signed-rank test [19]. Normal distribution of findings was evaluated using a Shapiro-Wilk test [20]. If a significant difference between sequences was identified, an additional Bonferroni-Holm post hoc test for multiple comparisons was conducted [21]. Significance was defined as *p*-values below 0.05.

Pathologies of all the evaluated structures of the cervical spine were separately noted by each reader. The inter-reader reliability for assessment of image quality and pathological findings was assessed using the intraclass correlation coefficient (ICC) [22]. For pathological findings, absolute values were compared. ICC values were interpreted as follows: below 0.5 indicated poor reliability, between 0.5 and 0.75 indicated moderate reliability, between 0.75 and 0.9 indicated good reliability, and values above 0.9 indicated excellent reliability [23]. The inter-modality agreement in the subgroup analysis with CT was measured using the Kappa statistics [24]. Kappa values between 0.41 and 0.60 were considered moderate, between 0.61 and 0.80 substantial, and above 0.81 almost perfect agreement [25].

All statistical analyses were performed using SPSS version 29.0 (IBM, Armonk, NY, USA).

## Results

**Fig. 1:**
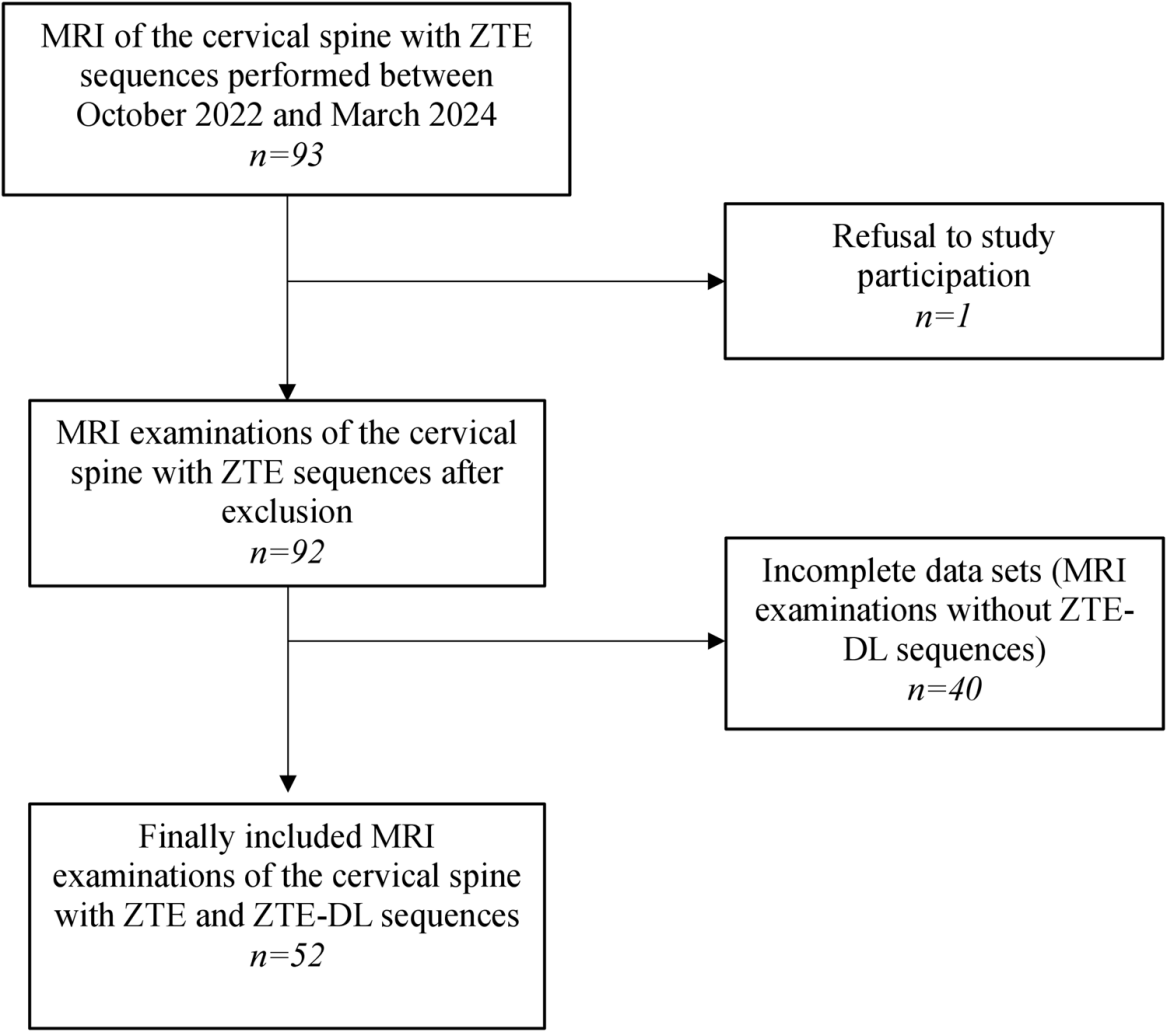
Flowchart of patients included in the study

Finally, 52 patients between 25 and 81 years of age (male *n* = 22, female *n* = 30) were included in the study. The clinical indications for MRI of the cervical spine encompassed cervical pain with radiculopathy (*n* = 14), unspecific cervical pain without radiculopathy (*n* = 26), a history of trauma involving the cervical spine (*n* = 9), rheumatoid arthritis (*n* = 1) and spondyloarthropathy (*n* = 2). A detailed description of patient selection is provided in the flowchart shown in Fig. 1.

In the subgroup analysis, in total twelve patients with CT and MRI examination with ZTE and ZTE-DL sequences of the cervical spine were included.

### Qualitative assessment of image quality and osseous structures

The mean overall image quality for ZTE and ZTE-DL sequences was 2.0 ± 0.7 and 3.2 ± 0.6, respectively, with significantly less artifacts in ZTE-DL sequences compared to ZTE sequences (*p* < 0.05).

The mean image quality of osseous structures was significantly better in ZTE-DL sequences compared to ZTE sequences both for cortical and trabecular bone (*p* < 0.05).

Detailed results of all evaluations are found in Table 3.

**Table 3:** Assessment of image quality and osseous structures in ZTE and ZTE-DL sequences

	ZTE sequences (mean ± SD)	ZTE-DL sequences (mean ± SD)	Wilcoxon signed-rank test (*p*-value)
Cortical bone at the level of atlantoaxial joint	2.6 ± 0.9	3.4 ± 0.8	< 0.001
Cortical bone at the level of C5	2.1 ± 0.6	3.1 ± 0.6	< 0.001
Trabecular bone at the level of C2	1.4 ± 1.0	2.2 ± 1.2	< 0.001
Trabecular bone at the level of C5	0.9 ± 1.0	1.9 ± 1.3	< 0.001
Overall image quality	2.0 ± 0.7	3.2 ± 0.6	< 0.001
Artifacts	0.8 ± 0.8	0.5 ± 0.7	< 0.001

The overall image quality of ZTE and ZTE-DL sequences was evaluated using a 5-point Likert scale (0—poor, 1—mild, 2—moderate, 3—good, and 4—perfect). Assessment of osseous structures was assessed separately for cortical bone at the level of atlantoaxial joint and C5 vertebra and for trabecular bone at the level of C2 and C5 vertebra

**Fig. 2:**
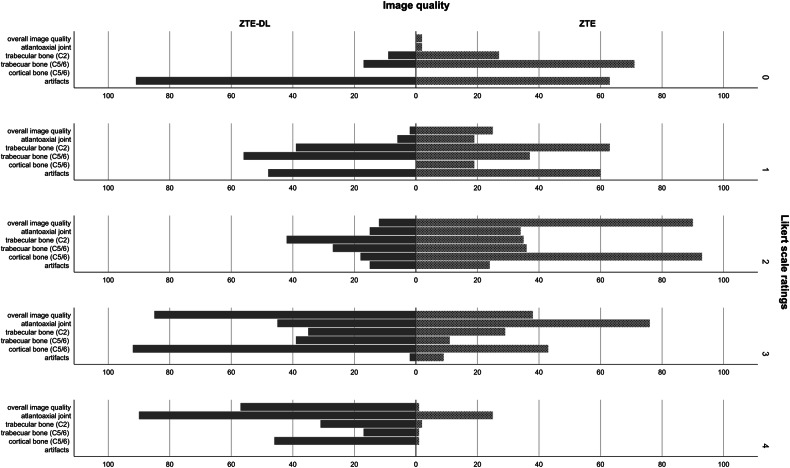
Scale ratings of all three readers for image quality and artifacts on ZTE and ZTE-DL sequences. On ZTE-DL sequences, overall image quality, delineation of trabecular and cortical bone, as well as image quality of the atlantoaxial joint were rated higher than on ZTE sequences, with the majority rated ‘3’ or higher. Artifacts were rated mostly 0 or 1 on ZTE-DL and ZTE sequences; overall fewer and less severe artifacts were observed on ZTE-DL sequences

Comparison of Likert scale ratings of all three readers regarding image qualities and artifacts ZTE and ZTE-DL sequences is found in Fig. 2.

### Quantitative assessment of image quality

The mean SNR ratio for bone at the level of C2 and C7 was 19.1 ± 7.6 and 15.1 ± 7.1 for ZTE sequences and 26.5 ± 13.0 and 19.5 ± 8.1 for ZTE-DL sequences, respectively, and was significantly higher for ZTE-DL sequences (*p* < 0.05).

**Fig. 3:**
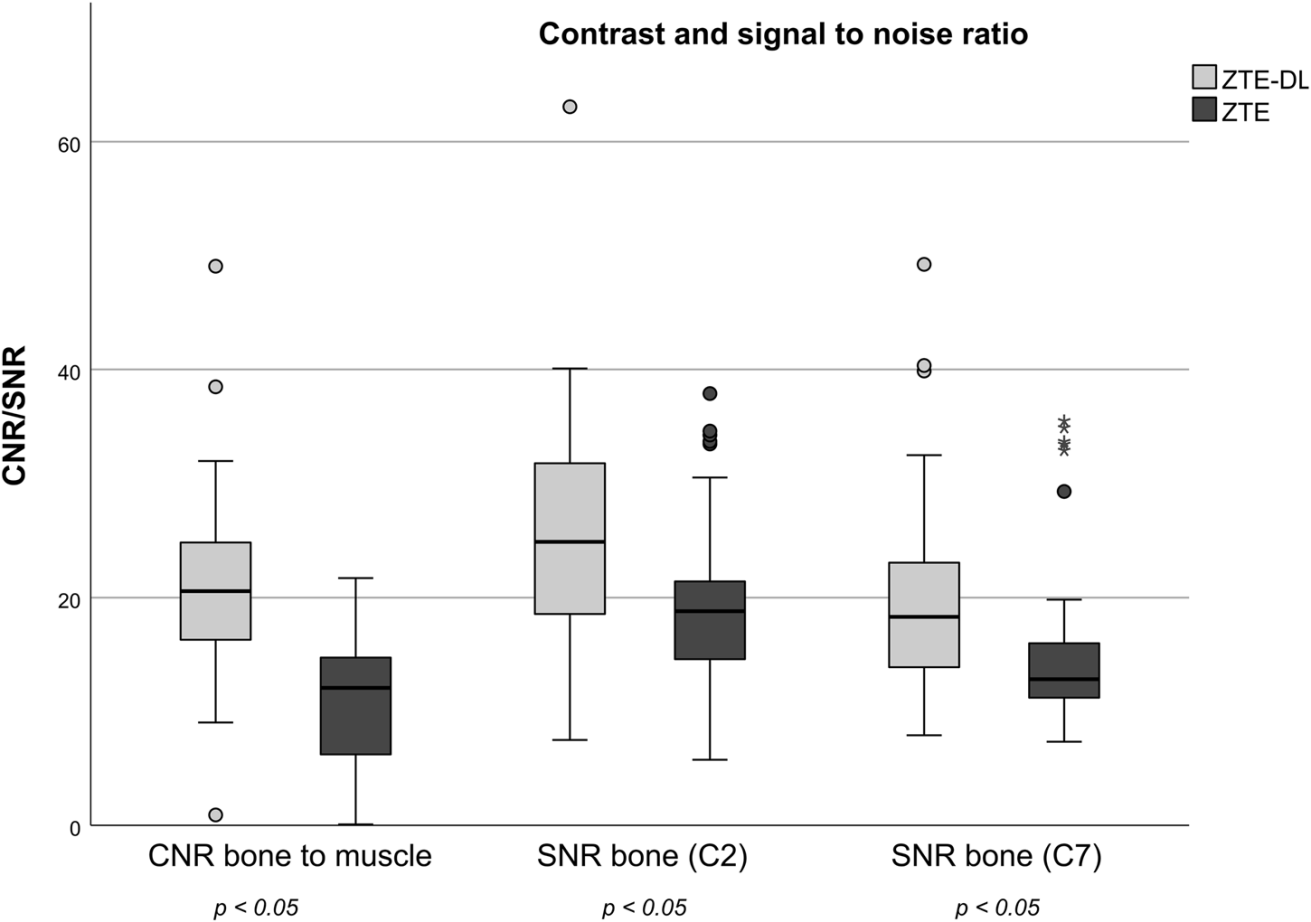
Box plots for SNR for bone and CNR for ZTE and ZTE-DL sequences. CNR and SNR showed significantly better results on ZTE-DL than on ZTE sequences. ROIs for measurements were placed in the vertebral bodies of C2 and C7 and in the adjacent cervical musculature

The mean CNR was significantly higher for ZTE-DL sequences compared to ZTE sequences (*p* < 0.05) with mean values of 11.0 ± 5.4 and 20.8 ± 7.9 for ZTE and ZTE-DL sequences, respectively. Figure 3 displays the box plots for SNR and CNR.

### Cervical structures and associated pathologies

The mean score in the evaluation of intervertebral space was 0.75 ± 0.03, 0.74 ± 0.03 and 0.64 ± 0.03 for ZTE, ZTE-DL and T2w sequences, respectively, while anterior osteophytes were rated as follows 0.80 ± 0.02, 0.79 ± 0.03 and 0.69 ± 0.03 and posterior osteophytes 0.58 ± 0.02, 0.63 ± 0.02 and 0.65 ± 0.02, respectively. Spinal canal and neural foraminal stenosis were scored in the following manner 0.55 ± 0.02, 0.57 ± 0.02 and 0.71 ± 0.02, and 0.74 ± 0.02, 0.68 ± 0.02 and 0.78 ± 0.02 for ZTE, ZTE-DL and T2w sequences, correspondingly. In terms of characterization of spinal canal and neural foraminal stenosis, the mean scores for ZTE, ZTE-DL and T2w sequences were 1.44 ± 0.05, 1.42 ± 0.05 and 0.90 ± 0.04, and 2.56 ± 0.02, 2.53 ± 0.02 and 2.02 ± 0.03, respectively.

**Fig. 4:**
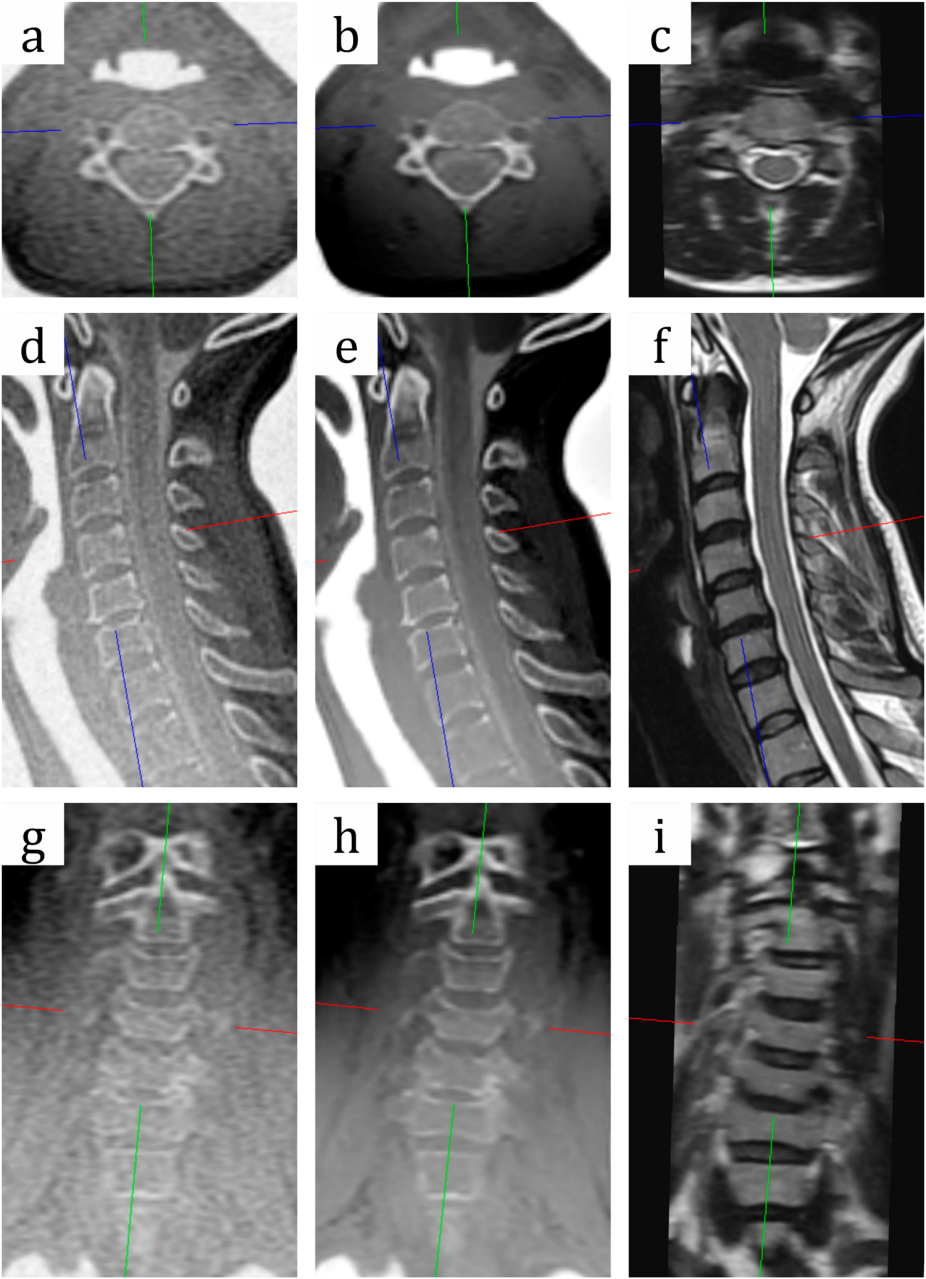
MR images of a 28-year-old female patient with cervical pain after trauma 3 months previously. Multiplanar reconstructions of the cervical spine with axial (**a**–**c**), sagittal (**d**–**f**), and coronal oblique (**g**–**i**) planes. ZTE sequences (**a**, **d**, **g**), ZTE-DL sequences (**b**, **e**, **h**) and T2w sequences (**c**, **f**, **i**) demonstrate minor spondylotic changes at the level of C5/6 with narrowing of intervertebral space and discrete anterior and posterior osteophytes at the endplates of C5/6

**Fig. 5:**
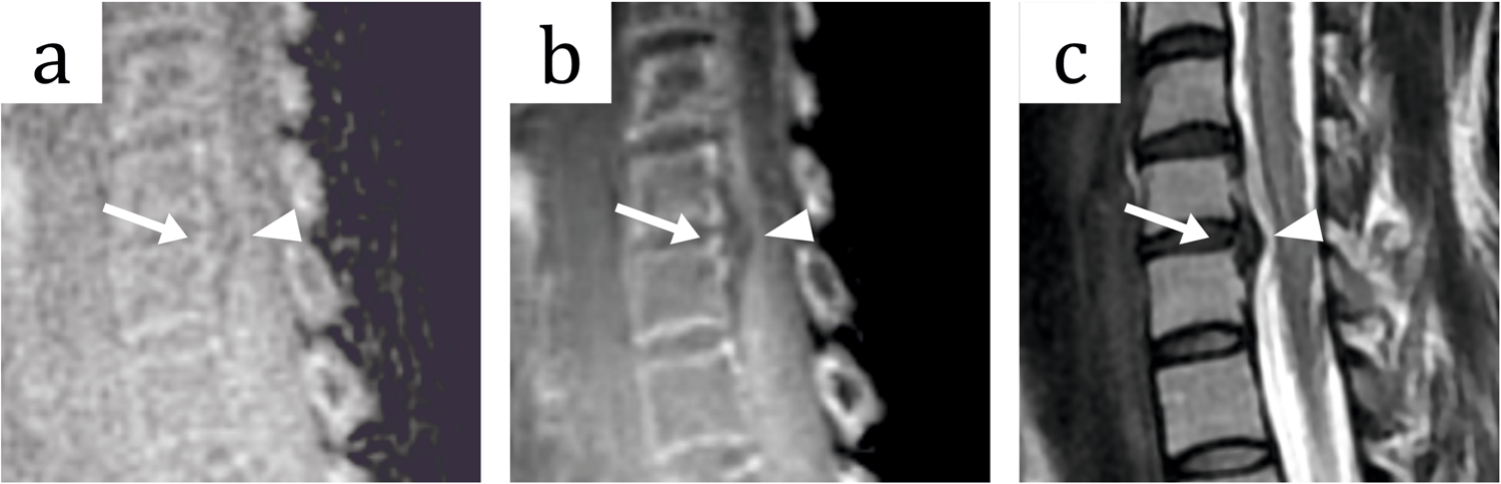
MR images of the cervical spine of a 39-year-old female patient with unspecific cervical pain without radiculopathy. **a** Sagittal zero-echo time (ZTE) sequence, (**b**) sagittal ZTE sequence with deep learning image reconstruction (ZTE-DL) and (**c**) sagittal T2-weighted (T2w) sequence show a disc herniation at the level C6/7 with a moderate spinal canal stenosis (arrowhead) with small osseous components (arrow). Although disc herniation and spinal canal stenosis are visible across all imaging sequences, the ZTE-DL sequences distinctly enhance the visualization of the osseous components

**Fig. 6:**
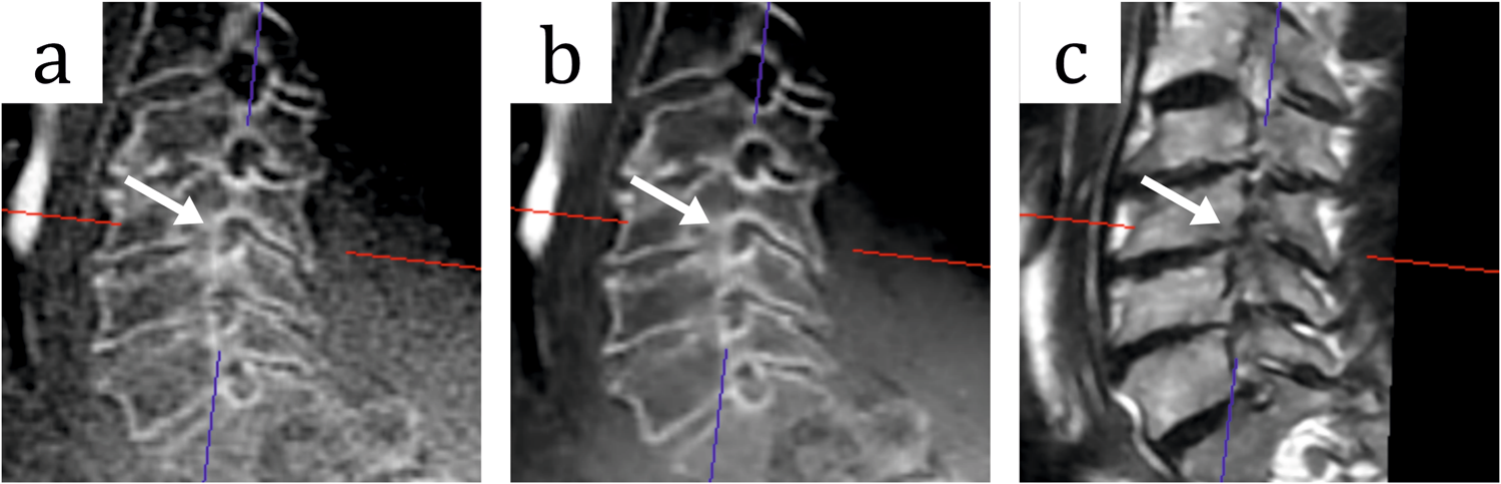
MR images of the cervical spine of a 50-year-old male patient with cervical pain with radiculopathy. **a** Sagittal zero-echo time (ZTE) sequence, **b** sagittal ZTE sequence with deep learning image reconstruction (ZTE-DL) and **c** sagittal T2-weighted (T2w) sequence show a moderate stenosis of the right neural foramen at the level C4/5 (arrow) with osseous components at the endplate of C4

Spinal canal stenosis due to osseous pathology (grade score > 2) was observed in 21.8%, 21.8% and 12.7% of patients with spinal canal stenosis in ZTE, ZTE-DL and T2w sequences, respectively, while involvement of bone in neural foraminal stenosis was identified in 46.5%, 43.2% and 37.1% of patients, respectively. Image examples of all sequences are shown in Fig. 4. Image examples with spinal canal and neural foraminal stenosis are shown in Figs. 5 and 6.

There was a significant difference between T2w and ZTE-DL sequences for the assessment of intervertebral space, anterior osteophytes, spinal canal stenosis, neural foraminal stenosis and in characterization of spinal canal and neural foraminal stenosis (*p*-values < 0.05) with ZTE-DL sequences providing a more accurate assessment of these structures. Furthermore, there was a significant difference between ZTE and ZTE-DL sequences for characterization of neural foraminal stenosis (*p*-values < 0.05) with better evaluation of osseous component of the stenosis in ZTE-DL sequences. The detailed results are found in Table 4.

**Table 4:** Evaluation of cervical structures and associated pathologies in T2w, ZTE and ZTE-DL sequences

		ZTE – ZTE-DL	Wilcoxon signed-rank test (*p*-value)	T2w - DL	Wilcoxon signed-rank test (*p*-value)
Intervertebral space	Negative ranks	44	0.434	115	**< 0.001**
	Positive ranks	54		38	
	Ties	682		627	
Anterior osteophytes	Negative ranks	57	0.807	120	**< 0.001**
	Positive ranks	62		46	
	Ties	661		614	
Posterior osteophytes	Negative ranks	85	**0.002**	95	0.242
	Positive ranks	47		113	
	Ties	648		572	
Spinal canal stenosis	Negative ranks	56	0.066	35	**< 0.001**
	Positive ranks	37		135	
	Ties	687		610	
Characterization of the spinal canal stenosis	Negative ranks	58	0.595	135	**< 0.001**
	Positive ranks	56		35	
	Ties	202		167	
Neural foraminal stenosis (right)	Negative ranks	39	**< 0.001**	74	**< 0.001**
	Positive ranks	73		109	
	Ties	668		597	
Characterization of the neural foraminal stenosis (right)	Negative ranks	31	**0.013**	130	**< 0.001**
	Positive ranks	51		44	
	Ties	250		136	
Neural foraminal stenosis (left)	Negative ranks	39	**< 0.001**	47	**< 0.001**
	Positive ranks	84		129	
	Ties	657		604	
Characterization of the neural foraminal stenosis (left)	Negative ranks	48	0.050	155	< 0.001
	Positive ranks	70		51	
	Ties	240		147	

Significant differences between sequences with *p*-values less than 0.05 appear in bold

### Subgroup analysis

The inter-modality agreement with CT for the assessment of cervical structures was almost perfect for ZTE and ZTE-DL sequences with Kappa values of 0.817 and 0.871, respectively. A detailed description of inter-modality agreements for all analyzed structures can be found in Table S1 in the Supplementary material. No statistically significant differences were found for the evaluation of any of the analyzed structures in ZTE and ZTE-DL sequences compared to the CT.

Evaluation of cervical structures and associated pathologies in ZTE and ZTE-DL sequences and in the CT may be found in Table S2 in the Supplementary material.

### Inter-reader agreements

There was a good inter-reader agreement for overall image quality of ZTE sequences and excellent for ZTE-DL sequences with ICC values of 0.894 and 0.904, respectively. There was a moderate inter-reader agreement for assessment of artifacts with ICC values of 0.728 and 0.749 for ZTE and ZTE-DL sequences, respectively.

In the overall assessment of cervical structures and associated pathologies, there was a good inter-reader agreement for ZTE, ZTE-DL and T2w sequences with ICC values of 0.894, 0.895 and 0.855, respectively. The detailed description of inter-reader agreements for all analyzed structures may be found in Table 5.

**Table 5:** Inter-reader agreement for evaluation of all analyzed structures of the cervical spine for ZTE, ZTE-DL and T2w sequences

	Inter-reader agreements		
	ZTE	ZTE-DL	T2w
Intervertebral space	0.893	0.867	0.887
Anterior osteophytes	0.873	0.895	0.886
Posterior osteophytes	0.834	0.829	0.777
Spinal canal stenosis	0.770	0.776	0.783
Characterization of the spinal canal stenosis	0.814	0.680	0.691
Neural foraminal stenosis (right)	0.847	0.857	0.836
Characterization of the neural foraminal stenosis (right)	0.292	0.307	0.439
Neural foraminal stenosis (left)	0.820	0.859	0.829
Characterization of the neural foraminal stenosis (left)	0.192	0.086	0.556
Overall	0.894	0.895	0.855

Inter-reader agreement was calculated using intraclass coefficient (ICC). ICC values under 0.5 were considered poor, between 0.5 and 0.75 moderate, between 0.75 and 0.9 good, and over 0.9 as an excellent reliability

## Discussion

To the best of our knowledge, this is the first study to evaluate image quality and additional benefit of ZTE and ZTE-DL sequences to a standard 3D T2w imaging sequence for imaging of osseous structures of cervical spine and associated pathologies. Both ZTE and ZTE-DL sequences provide a CT-like image impression, while the ZTE-DL sequences show significantly better image quality and fewer artifacts and serve as an important addition to standard T2w sequences for assessment and characterization of spinal canal and neural foraminal stenosis.

These results are concordant with previous studies on DL image reconstructions of MRI sequences for imaging of the different body regions, including knee and shoulder joint and lumbar spine [26–28], in which DL sequences showed significantly better image quality and anatomical conspicuity than standard sequences.

In our study, there was a significant difference between T2w and ZTE-DL sequences for the assessment of most cervical structures and in characterization of spinal canal and neural foraminal stenosis, leading to a better assessment of osseous components of the stenosis in ZTE-DL sequences. Higher grades and proportions of osseous components of spinal canal and neural foraminal stenosis were found when ZTE-DL sequences were evaluated together with T2w than when the stenosis was assessed on T2w sequences only.

These results are consistent with the study of Tran et al, in which ZTE sequences were added to a routine cervical spine MRI and showed a great potential in the evaluation of osseous structures [18]. While Tran et al investigated only ZTE sequences for imaging degenerative changes in the cervical spine, we also included ZTE-DL sequences alongside conventional ZTE sequences. This approach resulted in better image quality and more detailed visualization of both trabecular and cortical bone in ZTE-DL sequences, with reduced image noise and artifacts compared to ZTE sequences. Additionally, we quantitatively assessed the image quality of ZTE and ZTE-DL sequences by placing regions of interest in the C2 and C7 vertebrae to measure signal homogeneity throughout the images. These measurements demonstrated significantly better contrast-to-noise and signal-to-noise ratios for ZTE-DL sequences compared to ZTE sequences.

Our study outlines the importance of ZTE-DL sequences as addition to a regular MRI of cervical spine, especially in patients with spinal canal or neural foraminal stenosis, because they enable a detailed characterization of involvement of bone in these pathologies. An osseous component was observed in 21.8% of patients with spinal canal stenosis in ZTE and ZTE-DL sequences, and only in 12.7% of patients in T2w sequences with a significant difference between T2w only and when ZTE-DL sequences were added to the routine protocol. This might be due a relatively small size of the osseous components, which may be identified in the CT-like images of ZTE and ZTE-DL sequences but are hardly visible in conventional T2w sequences (Fig. 5). The assessment of bone involvement in patients with neural foraminal stenosis was identified in 46.5% and 43.2% in ZTE and ZTE-DL sequences, however only in 37.1% of patients in T2-weighted sequences with a significant difference between T2w and ZTE-DL sequences. Enhanced certainty in assessing the extent of bone and soft tissue involvement in spinal canal and neural foraminal stenosis was also observed in the study of Tran et al in conventional ZTE sequences [18]. In our study, the addition of ZTE-DL sequences resulted in a significantly better image quality and improved depiction of osseous structures and therefore should be considered in a clinical scenario rather than conventional ZTE sequences.

Conventionally, CT scans are performed as an adjunct to an MRI scan in preoperative planning of cervical spine surgery when significant osseous changes are suspected [29–32]. However, ionizing radiation of CT is still an important limitation, and therefore alternative radiation-free examinations are highly desirable. The potential of ZTE sequences to replace CT scans has already been shown in different studies, for example, in imaging of extremities, spine and head and neck [10, 33–35]. Moreover, the application of ZTE sequences has been demonstrated as efficacious in the preoperative planning of neurosurgical procedures with a good correlation with CT [36]. Our study highlights that ZTE-DL sequences present a reliable, radiation-free option for imaging osseous pathologies in the cervical spine, particularly for assessing bone involvement in spinal canal and neural foraminal stenosis. Given the strong inter-reader agreement of three readers observed in our study for evaluating diverse pathological findings in the cervical spine using both ZTE and ZTE-DL sequences, these results indicate their promising role in preoperative imaging. Further studies addressing this potential utility are therefore needed.

Our study has several limitations. First, we did not correlate all findings detected in ZTE and ZTE-DL sequences with CT. To address this, we performed a subgroup analysis involving twelve patients who had both CT and MRI of the cervical spine. This analysis revealed almost perfect inter-modality agreement between CT and both ZTE and ZTE-DL sequences in assessing cervical structures. Previous studies have also demonstrated a strong correlation between ZTE sequences and CT for evaluating the cervical spine and associated pathologies, with high diagnostic accuracy in identifying and characterization of stenosis of the spinal canal and neural foramen [18, 33]. The primary aim of our study was to examine noninvasive MRI sequences that offer CT-like imaging characteristics. Therefore, we opted not to acquire additional CT images but instead conducted a subgroup analysis. Second, a relatively small population of 52 patients was included in our study. Third, we did not analyze the application of ZTE-DL sequences for imaging of traumatic cervical spine injuries in acute trauma setting. This topic would be interesting to explore and analyze in greater detail in future studies, particularly in patients with brain injuries where MRI is routinely performed. Lastly, although all sequences were anonymized and all sequence identifiers removed, the ZTE-DL sequences could still be differentiated from ZTE in some cases due to their significantly smoother image impression and fewer artifacts.

In conclusion, ZTE-DL sequences offer a superior depiction of trabecular and cortical bone in the cervical spine compared to ZTE sequences, demonstrating higher image quality and reduced artifacts. ZTE and ZTE-DL sequences enable reliable assessment of various cervical spine pathologies, particularly for characterizing of osseous components in spinal canal and neural foraminal stenosis and result in better characterization of the stenosis than in T2w sequences only. Therefore, incorporating ZTE-DL sequences into routine cervical spine MRI protocols as an addition to T2w sequences warrants consideration in clinical practice.

## Supplementary information

ELECTRONIC SUPPLEMENTARY MATERIAL

## Data Availability

All data supporting the results (including all MRI images of the patients included in the study, patients’ demographics, excel tables with all information and according statistical results) reported in this article can be found on the central disc storage of the Department of Diagnostic and Interventional Radiology at the University Hospital Zurich.

## Abbreviations

CNN: Convolutional neural networks
CNR: Contrast-to-noise ratio
ICC: Intraclass coefficient
ROI: Regions of interest
SD: Standard deviation
SNR: Signal-to-noise ratio
ZTE: Zero-echo time sequences
ZTE-DL: Deep learning-based reconstructions of zero-echo-time sequences
