# Supplementary material for: Deep learning reconstruction of zero-echo time sequences to improve visualization of osseous structures and associated pathologies in MRI of cervical spine
Source: Insights Imaging. 2025 Jan 29;16:29. doi: 10.1186/s13244-025-01902-0 (PMC11780046; doi:10.1186/s13244-025-01902-0)
Supplement: Supplementary file 1 — ELECTRONIC SUPPLEMENTARY MATERIAL [file 13244_2025_1902_MOESM1_ESM.pdf]

Deep learning reconstruction of zero-echo time sequences to improve visualization of osseous structures and associated pathologies in MRI of cervical spine

ELECTRONIC SUPPLEMENTARY MATERIAL

Table S1 Inter-modality agreement for evaluation of all analyzed structures of the cervical spine for ZTE, ZTE-DL, and CT.

Intermodality agreements			
	ZTE - CT	ZTE - DL - CT	ZTE - ZTE - DL
Intervertebral space	0.833	0.875	0.937
Anterior osteophytes	0.758	0.789	0.825
Posterior osteophytes	0.742	0.891	0.854
Spinal canal stenosis	0.907	0.951	0.948
Characterization of the spinal canal stenosis	0.587	0.794	0.633
Neural foraminal stenosis (right)	0.843	0.889	0.889
Characterization of the neural foraminal stenosis (right)	0.529	0.772	0.441
Neural foraminal stenosis (left)	0.810	0.863	0.897
Characterization of the neural foraminal stenosis (left)	0.448	0.551	0.764
Overall	0.817	0.871	0.880

The inter-modality agreement in the subgroup analysis with CT was measured using the Kappa statistics. Kappa values between 0.41-0.60 were considered moderate, between 0.61-0.80 substantial, and above 0.81 almost perfect agreement.

Table S2. Evaluation of cervical structures and associated pathologies CT, ZTE, and ZTE-DL sequences.

		**ZTE – DL - CT**	**Wilcoxon signed rank test (*p* value)**	**ZTE - CT**	**Wilcoxon signed rank test (*p* value)**
Overall	Negative ranks	20	0.086	24	0.899
	Positive ranks	11		25	
	Ties	352		369	
Intervertebral space	Negative ranks	1	0.317	2	0.414
	Positive ranks	3		4	
	Ties	51		54	
Anterior osteophytes	Negative ranks	4	0.705	3	0.317
	Positive ranks	3		6	
	Ties	48		51	
Posterior osteophytes	Negative ranks	3	0.083	5	0.763
	Positive ranks	0		3	
	Ties	52		52	
Spinal canal stenosis	Negative ranks	1	0.317	2	0.157
	Positive ranks	0		0	
	Ties	54		58	
Characterization of the spinal canal stenosis	Negative ranks	1	0.655	2	0.458
	Positive ranks	1		2	
	Ties	11		9	
Neural foraminal stenosis (right)	Negative ranks	2	0.564	3	0.655
	Positive ranks	1		2	
	Ties	52		55	
Characterization of the neural foraminal stenosis (right)	Negative ranks	2	0.157	0	0.102
	Positive ranks	0		3	
	Ties	17		19	

Neural foraminal stenosis (left)	Negative ranks	1	0.317	2	0.414
	Positive ranks	3		4	
	Ties	51		54	
Characterization of the neural foraminal stenosis (left)	Negative ranks	5	**0.025**	5	0.102
	Positive ranks	0		1	
	Ties	16		17	

Significant differences between sequences with p-values less than 0.05 appear in bold.
